# Effects of a Two-Year Lifestyle Intervention on Intrahepatic Fat Reduction and Renal Health: Mitigation of Inflammation and Oxidative Stress, a Randomized Trial

**DOI:** 10.3390/antiox13070754

**Published:** 2024-06-21

**Authors:** Maria Magdalena Quetglas-Llabrés, Margalida Monserrat-Mesquida, Cristina Bouzas, Silvia García, David Mateos, Miguel Casares, Cristina Gómez, Lucía Ugarriza, Josep A. Tur, Antoni Sureda

**Affiliations:** 1 Centro de Investigación Biomédica en Red de Fisiopatología de la Obesidad y Nutrición (CIBEROBN), Instituto de Salud Carlos III, 28029 Madrid, Spain; m.quetglas@uib.es (M.M.Q.-L.); margalida.monserrat@uib.es (M.M.-M.); cristina.bouzas@uib.es (C.B.); silvia.garcia@uib.es (S.G.); davidfrom13@gmail.com (D.M.); luciaugarriza@gmail.com (L.U.); antoni.sureda@uib.es (A.S.); 2 Research Group on Community Nutrition & Oxidative Stress, University of Balearic Islands-IUNICS, 07120 Palma de Mallorca, Spain; cristina.gomez@ssib.es; 3 Health Research Institute of the Balearic Islands (IdISBa), 07120 Palma de Mallorca, Spain; 4 Radiodiagnosis Service, Red Asistencial Juaneda, 07011 Palma de Mallorca, Spain; casaresmiguel@gmail.com; 5 Clinical Analysis Service, University Hospital Son Espases, 07198 Palma de Mallorca, Spain; 6 C.S. Camp Redó, IBSalut, 07010 Palma de Mallorca, Spain

**Keywords:** MAFLD, renal dysfunction, intrahepatic fat content, lifestyle change, oxidative state, inflammation, intervention, fatty liver

## Abstract

Metabolic-associated fatty liver disease (MAFLD) is the most common chronic liver disease observed in clinical practice worldwide. This disorder has been independently associated with an increased risk of developing chronic kidney disease (CKD). The aim of this study was to evaluate whether a 2-year intervention based on a Mediterranean diet (MedDiet) and physical activity focussed on reducing intrahepatic fat contents (IFC) was associated with a decreased risk of CKD. Forty adults (50% women) residing in Mallorca, aged 48 to 60 years, diagnosed with MAFLD were recruited. Participants were divided into two groups based on whether they improved IFC measured by nuclear magnetic resonance. Anthropometric and clinical parameters improved in responders, including reduced weight, body mass index (BMI), and waist circumference. Only responders showed improvements in lipid profile and liver enzymes. Haematological parameters showed favourable changes in both groups. Oxidative stress and inflammatory biomarkers differed between groups. Responders had lower plasma interleukine-18 (IL-18) levels, but higher erythrocyte malonaldehyde (MDA) levels. Non-responders showed increased erythrocyte catalase and superoxide dismutase activity. After 2 years, non-responders had higher serum creatinine, Modification of Diet in Renal Disease (MDRD), and Chronic Kidney Disease Epidemiology Collaboration (CKD-EPI) levels, while responders showed reductions in these parameters together with uric acid and urine albumin-to-creatinine ratio (UACR). Positive correlations were found between changes in IFC and kidney injury biomarkers, including MDRD and serum creatinine levels. In conclusion, a healthy diet based on the Mediterranean dietary pattern and lifestyle promotes significant improvements in parameters related to cardiovascular, hepatic, and renal health.

## 1. Introduction

The term non-alcoholic fatty liver disease (NAFLD) refers to excessive fat accumulation in the liver, characterized by the presence of steatosis in more than 5% of hepatocytes, and often associated with insulin resistance [1]. NAFLD has been progressively replaced by the term metabolic-associated fatty liver disease (MAFLD) to highlight the importance of metabolic abnormalities, as it better aligns with the evolving understanding of disease mechanisms [2]. MAFLD has surged to the top of the liver disease spectrum globally, affecting an estimated 38% of the global population. Although cirrhosis and hepatocellular carcinoma are rare outcomes for MAFLD patients, the growing number of people at risk of these dangerous conditions is cause for concern. Furthermore, the tendency of MAFLD to manifest at younger ages raises serious implications for long-term health outcomes [3].

Oxidative stress and inflammation play crucial roles in the pathophysiology of MAFLD [4]. Elevated levels of reactive oxygen species (ROS) can instigate lipid peroxidation, damaging cellular membranes, key metabolic proteins, and nucleic acids [5]. In conditions of overfeeding, such as in MAFLD, the liver’s capacity to store triglycerides is overwhelmed, leading to the accumulation of saturated fatty acids, which impair cellular function. Excessive fatty acid oxidation increases ROS production and lipotoxicity, causing further cellular damage and oxidative stress [6]. This cascade is linked to elevated markers of oxidative damage, activation of Kupffer cells and proinflammatory pathways, and recruitment of immune cells from circulation [7]. Chronic inflammation often stems from unresolved acute inflammation, commonly seen in metabolic disorders like diabetes, obesity, metabolic syndrome, and MAFLD, as well as in certain cancers characterized by subclinical inflammation [8]. The presence of inflammation associated with non-alcoholic steatohepatitis (NASH) indicates a higher risk of fibrosis and disease progression in MAFLD patients [9]. Therefore, monitoring the oxidative and inflammatory state over time is important, in addition to the analysis of the more clinical parameters of the pathology.

Until recently, pharmacotherapy for MAFLD or NASH was considered an unmet clinical need, with no drugs receiving FDA approval specifically for NASH [10]. However, with the recent approval of Rezdiffra (resmetirom) by the FDA, a significant milestone has been achieved in addressing this gap [11]. Nonetheless, lifestyle intervention remains a cornerstone of treatment for MAFLD, emphasizing the importance of holistic management approaches. A meta-analysis published in 2021 revealed that following a Western diet might raise the risk of MAFLD by 56%, suggesting that dietary pattern is linked to the disease’s risk. In contrast, a Mediterranean diet (MedDiet) could lower this risk by 23% [12]. In a previous study, we evidenced that increasing the adherence to the MedDiet improved the main MAFLD features, including the intrahepatic fat and plasma liver enzymes, but also improved intestinal permeability [13]. Moreover, a systematic review established the beneficial effects of the MedDiet and physical activity in relation to MAFLD as an effective treatment to prevent and even reverse the pathology. This effect is particularly significant when combining both interventions, being more effective than physical activity or diet alone [14]. It has been demonstrated in the last ten years that MAFLD may influence not only the prognosis connected to the liver but also function as a separate risk factor for a number of chronic illnesses, such as cardiovascular disease (CVD) [15], chronic kidney disease (CKD) [16], and extra-hepatic cancers [17].

CKD is defined as a glomerular filtration rate (GFR) <60 mL/min per 1.73 m^2^ or markers of kidney damage (Albuminuria ≥30 mg/g, urinary sediment abnormality, electrolyte or other abnormality due to tubular disorder, abnormalities on histology, structural abnormalities detected by imaging, and history of kidney transplantation), or both, of at least 3-month duration [18]. It is important to note that in people with obesity and metabolic disorders, whether they are diabetic or not, CKD often begins with a period of excessive filtering in the kidneys, known as glomerular hyperfiltration [19,20]. This hyperfiltration phase is associated with an increased risk of rapid decline in renal function and leakage of albumin in urine [21]. Studies have shown that reducing hyperfiltration through drugs that target the renin–angiotensin–aldosterone system (RAAS) or weight loss can exert protective effects on the kidneys in patients with type 2 diabetes mellitus (T2DM), slowing down the decline in GFR over time, similar to the rate observed in healthy aging adults [21,22]. This highlights the importance of early detection and management of renal complications associated with metabolic disorders. Notably, individuals with MAFLD exhibit a higher prevalence of CKD and abnormal albuminuria compared to those without MAFLD [23]. It was estimated that 20–25% of individuals with fatty liver also suffer from CKD [24]. Studies consistently reported a correlation between the severity of MAFLD and the progression of CKD, which is further exacerbated by shared risk factors such as obesity, diabetes, and hypertension [23]. A recent meta-analysis, comprising 13 longitudinal studies with over 1.2 million individuals, highlighted a significant association between MAFLD and a high risk of incident CKD, with a hazard ratio of 1.43 (95% confidence interval 1.33–1.54), over a median follow-up period of nearly 10 years [25].

The specific pathogenic mechanisms connecting MAFLD and CKD are not completely comprehended. The strong interconnection of each disease with abdominal obesity and insulin resistance makes it difficult to pinpoint the primary causal factors behind the elevated risk of CKD in MAFLD patients [26]. Some studies proposed that the enlarged or inflamed adipose tissue, which releases free fatty acids and proinflammatory adipocytokines, thereby inducing systemic insulin resistance, plays a significant role in both conditions. Moreover, liver fibrosis (and consequently stiffness) characteristic of MAFLD may also contribute to the development of albuminuria and CKD. Research pointed out that MAFLD and its advancement to fibrosis coincided with the increased expression of pro-fibrogenic cytokines (including fibroblast growth factor-21 (FGF-21) and transforming growth factor-β (TGF-β)), which could potentially impact kidney function, alongside a state of low-grade inflammation and a pro-thrombotic environment [27].

Considering the relationship between MAFLD and CKD, the aim of this study was to evaluate whether a nutritional intervention based on a hypocaloric MedDiet combined with the promotion of physical activity, specifically designed for patients with MAFLD, can reduce intrahepatic fat content (IFC) and impact kidney function. Additionally, biomarkers of oxidative and inflammatory status, which are involved in the pathogenesis of both disorders, were also analysed.

## 2. Methods

### 2.1. Study Design

The two-year prospective randomized controlled trial introduced a personalized 2-year intervention with MedDiet and physical activity, with the aim of preventing and reversing MAFLD among patients with overweight or obesity and metabolic syndrome (MetS). The study adhered to the ethical principles outlined in the Declaration of Helsinki, and each step of the process was authorized by the Ethics Committee of the Balearic Islands (CEIC-IB2251/14PI). Informed consent was obtained from all participants after they were informed about the study’s objectives and potential outcomes. The study was registered on ClinicalTrials.gov with the registration number NCT04442620 [28].

The following criteria were necessary for inclusion: ages between 40 and 60, a body mass index (BMI) between 27 and 40 kg/m^2^, exhibition of at least three of the five MetS features listed in the International Diabetes Federation (IDF) [29] consensus, and MAFLD diagnosed by magnetic resonance imaging (MRI). The exclusion criteria utilized encompassed individuals with a history of cardiovascular disease; liver conditions excluding NAFLD; viral, autoimmune, and genetic liver ailments; recent or ongoing malignancy; prior bariatric surgery; untreated depression or anxiety; excessive alcohol or drug consumption; pregnancy; primary endocrine disorders excluding hypothyroidism; recent use of weight loss medications; or ongoing steroid therapy or those unable or unwilling to provide informed consent or engage in communication with the study team.

Following their inclusion in the trial, individuals were randomized into one of three groups as follows:

Conventional diet (CD) group: Participants adhered to the guidelines set by the American Association for the Study of Liver Diseases (AASLD), aiming for a weight loss of 3–5% to ameliorate steatosis and 7–10% to enhance most histopathological features of NASH. This was achieved through energy restrictions in alignment with the general dietary guidelines of the U.S. Department of Health and Human Services and the U.S. Department of Agriculture, consisting of 20–35% fat, 10–35% protein, and 45–65% carbohydrates.

MedDiet with high meal frequency (MD-HMF) group: This group followed a MedDiet with macronutrient distribution of 40–45% carbohydrates (50–70% being low glycaemic and fibre-rich), 30–35% fat, and 25% protein. Previous studies have shown that this diet reduces fat mass and total body weight and enhances oxidative status in individuals with metabolic syndrome [30]. Daily caloric intake was divided into seven meals, with the highest-calorie meals consumed early in the day.

MedDiet with physical activity (MD-PA) group: Participants in this group consumed an energy-restricted MedDiet with four to five meals per day, including snacks. Their total caloric intake consisted of 35–40% fat (8–10% saturated fatty acids, >20% monounsaturated fatty acids, >10% polyunsaturated fatty acids, and <300 mg/day cholesterol), approximately 20% protein, and 40–45% carbohydrates (mainly low glycaemic index). Sodium chloride intake was limited to 6 g/day (2.4 g sodium), and dietary fibre was set at a minimum of 30–35 g/day.

Participants in the CD and MD-HMF groups were instructed to walk at least 10,000 steps daily, while the MD-PA group additionally engaged in a 35 min interval training session three times a week. All three nutritional interventions involved a 25–30% reduction in baseline caloric intake and an increase in energy expenditure by 5.7 kcal/kg of body weight.

**Figure 1:**
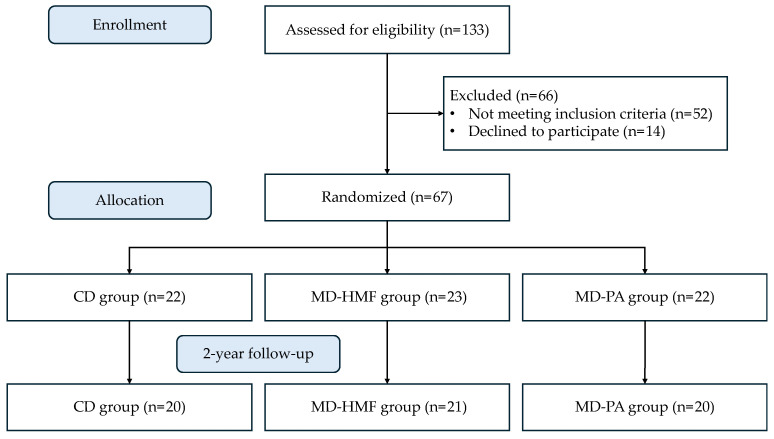
Flow diagram of the study.

There were 133 patients examined for eligibility; 52 did not match the requirements, and 14 declined to participate. Finally, 67 patients were assigned in a 1:1:1 ratio to one of the three therapeutic groups for two years. The overall sample size was 61, with a subgroup of 40 subjects examined for oxidative and inflammatory data (Figure 1).

### 2.2. Participants

In a previously published work, we observed that the improvement in IFC and liver stiffness was similar in the three groups [31]. Within each group, the subjects who better adhered to the recommendations were those who presented the greatest improvements. Thus, for the present work, a subsample of 40 patients was selected (13 from the CD group, 14 from the MD-HMF group, and 13 from the MD-PA group) based on whether they improved their IFC after the intervention. Consequently, the participants were distributed into two groups: those who managed to reduce their IFC after 24 months of lifestyle intervention (CD: 5, MD-HMF: 7, MD-PA: 8; n = 20) and those who did not achieve this reduction (CD: 8, MD-HMF: 7, MD-PA: 5; n = 20).

### 2.3. Anthropometrics and Clinical Assessment

Accurate and consistent anthropometric measurements were conducted by trained professional dieticians who underwent identical and rigorous training to minimize the impact of interobserver coefficients of variation. Body weight, determined without shoes, was assessed using a segmental body composition analyser (Tanita BC-418, Tanita, Tokyo, Japan), with a deduction of 0.6 kg for light clothing. Height measurements were taken with a mobile anthropometer (Seca 214, SECA Deutschland, Hamburg, Germany), ensuring the patient’s head was in the Frankfort Horizontal Plane position. BMI (kg/m^2^) was then calculated based on these measurements. While the subjects were standing straight, their waist circumference was measured twice using an anthropometric tape, midway between the last rib and the iliac crest. Blood pressure was measured in triplicate while the patient was in a sitting position, using a validated semi-automatic oscillometer (Omron HEM, 750CP, Hoofdrop, The Netherlands). 

### 2.4. Steatosis and Fibrosis Measure

Intrahepatic fat contents (IFCs) were measured with 1.5-T magnetic resonance imaging (MRI) (Signa Explorer 1.5T, General Electric Healthcare, Chicago, IL, USA) by using a 12-channel phased-array coil [32]. Abdominal MRI allows quantification of the liver fat as a mean percentage, and a mean IFC ≥ 6.4% was established as clinically relevant.

Evaluation of liver fibrosis by measurement of liver stiffness was determined by transient elastography using FibroScan^®^ (Echosens, Paris, France). Patients were examined lying down in a resting respiratory position, with the right arm elevated above the head for optimal intercostal access. Measurements were obtained at least 1.5 to 2.0 cm deep to the liver capsule and at a depth of less than 4 cm to the skin surface to avoid the reverberation artefact. Sonographers placed a region of interest in the hepatic parenchyma, avoiding large blood vessels. Transient elastography was performed after finding an adequate liver portion free of large vessels. Repeated shots were performed until 10 valid measurements were obtained. The success rate was calculated as the number of valid measurements divided by the total number of measurements, and a success rate of at least 60% was considered reliable. 

### 2.5. Blood and Urine collection

At each visit, venous blood from the antecubital vein and single-spot urine samples were collected after an overnight fasting period.

Fasting glucose, glycated haemoglobin A1c (HbA1c), triglycerides, high-density lipoprotein cholesterol (HDL-c), low-density lipoprotein cholesterol (LDL-c), total cholesterol, aspartate aminotransferase (AST), alanine aminotransferase (ALT), gamma-glutamyl transferase (GGT), and c-reactive protein (CRP) were measured in serum on the Abbott ARCHITECT c16000 employing commercial kits (Abbott Diagnostics, Lake Bluff, IL, USA) in Son Espases Hospital’s clinical laboratory (Palma de Mallorca, Spain). The serum ferritin was determined in an Abbott ARCHITECT i2000 (Abbott Diagnostics, Lake Bluff, IL, USA) using a chemiluminescence assay. Haematological parameters and cell counts were evaluated utilizing an automated flow cytometer analyser, specifically the Technion H2 (Bayer, Frankfurt, Germany) VCS system. In our laboratory, to obtain plasma and erythrocytes samples, fresh blood collected in suitable vacutainers with ethylenediaminetetraacetic acid (EDTA) as an anticoagulant was centrifugated at 1700× *g* 15 min at 4 °C. 

Urine samples were collected in clean, dry containers during the first morning void in rest settings. Albumin and creatinine levels were determined using a modified Jaffe technique on an Abbott ARCHITECT c16000 (Abbott Diagnostics, Lake Bluff, IL, USA) in Son Espases Hospital. All urine biochemical parameter findings were normalized by creatinine levels. Urinary albumin excretion, expressed as urine albumin-to-creatinine ratio (UACR), and estimate GFR were calculated using the Modification of Diet in Renal Disease (MDRD) formula [33] and the Chronic Kidney Disease Epidemiology Collaboration (CKD-EPI) formula [34].

### 2.6. Lifestyle: Mediterranean Diet and Physical Activity

Total energy intake was measured utilizing a validated semi-quantitative 143-item FFQ on dietary consumption [35]. A validated questionnaire with 17 diet-related questions was used to assess the participants’ compliance with the MedDiet pattern with an energy restriction [36]. Greater adherence to the MedDiet was indicated by a higher score on this questionnaire. The maximal oxygen uptake (VO_2_ max) of the patients was measured with the Chester step test [37].

### 2.7. Enzymatic Determinations

All enzymatic activities were determined in erythrocytes using a Shimadzu UV-2100 spectrophotometer (Shimadzu Corporation, Kyoto, Japan) at 37 °C. Catalase (CAT) activity was analysed at 240 nm by the spectrophotometric method of Aebi based on the decomposition of H_2_O_2_ [38]. Superoxide dismutase (SOD) activity was determined by an adaptation of McCord and Fridovich’s method at 550 nm [39].

### 2.8. Protein Carbonyl

Protein carbonyl derivatives were identified using an OxiSelect^TM^ Protein Carbonyl Immunoblot Kit (CELL BIOLABS^®^, San Jose, CA, USA) in accordance with the instructions provided. The Bradford technique was used to determine the total protein levels in the samples, with a commercial reagent (Merck Life Science S.L.U., Madrid, Spain). Using the dot blot technique (Bio-Rad, Hercules, CA, USA), 10 μg of protein was transferred to a nitrocellulose membrane and treated with 2,4-dinitrophenylhydrazine (DNPH). The membrane was then treated with the DNPH-specific primary antibody (1:1000). This was followed by incubation with goat antirabbit IgG (1:5000). The immunoblot was then developed using an enhanced chemiluminescence kit (Immun-Star Western C Kit reagent, Bio-Rad Laboratories, Hercules, CA, USA). Finally, the protein carbonyl bands were quantified using the image analysis software Quantity One1-D (Bio-Rad Laboratories, Hercules, CA, USA).

### 2.9. Malondialdehyde Assay

Malonaldehyde as a marker of lipid peroxidation was analysed in erythrocytes of all participants by a specific colorimetric assay kit (Sigma-Aldrich Merck^®^, St. Louis, MO, USA), where the absorbance was measured at 586 nm following the manufacturer’s instructions.

### 2.10. Immunoassay Kits

Interleukin-18 (IL-18) and TGF-β1 levels were determined in plasma using a Human IL-18 ELISA kit (Elabscience^®^, Elabscience Biotechnology, Houston, TX, USA) and a Human TGF-β1 ELISA kit (Elabscience^®^, Elabscience Biotechnology, Houston, TX, USA).

Interleukine-6 (IL-6) and tumour necrosis factor alpha (TNFα) levels were determined in plasma using Human Custom ProcartaPlex^TM^ (Invitrogen by Thermo Fisher Scientific, Bender MedSystems GmbH, Vienna, Austria). 

Cystatin C (CysC), neutrophil gelatinase-associated lipocalin (NGAL), and α-1-microglobulin (α1m) levels were determined in urine using Human Custom ProcartaPlex^TM^ Human, NHP, and Canine Mix & Match Panels (Invitrogen by Thermo Fisher Scientific, Bender MedSystems GmbH, Vienna, Austria). 

All immunoassay kits were used following the supplier’s guidelines for use.

### 2.11. Statistical Methods

Statistical analysis was carried out with the Statistical Package for Social Sciences (SPSS v.29, IBM Software Group, Chicago, IL, USA). Variables were presented as mean ± standard deviation (SD), considering *p* < 0.05 as statistically significant. The Kolmogorov–Smirnov test was used to assess the normality of data. Two-way analysis of variance (ANCOVA) after adjustment for age and gender was used to check the significance of data. The Bonferroni post hoc test was carried out when significant differences were found between groups. 

## 3. Results

**Figure 2:**
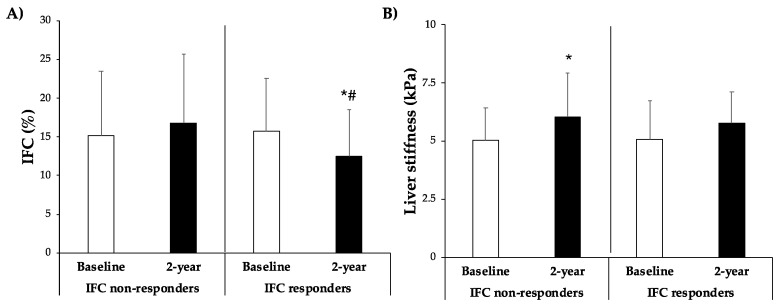
(**A**) Intrahepatic fat contents (IFCs) expressed as percentage of fat in the liver and (**B**) liver stiffness expressed as kPa at baseline and 2 years stratified by improvement of IFC group. Results are presented as mean ± SD. Two-way analysis of co-variance (ANCOVA) after adjustments by age and sex. * Difference in means between participants over time (baseline and 2 years). # Difference in means between groups (IFC responders and IFC non-responders). Data points are significant when *p* < 0.05.

Figure 2 depicts the IFC and liver stiffness of the individuals at the start of the trial and after two years of intervention. Initially, there were no differences between both groups, with an IFC of 15.1 ± 8.4 and 15.7 ± 6.9 for IFC non-responders and IFC responders, respectively. Following the 2-year intervention, the IFC changed to 16.8 ± 8.9 and 12.5 ± 6.0, respectively. Participants who achieved a reduction in their IFC after a two-year intervention did so significantly, attaining a lower IFC compared to the other group. Participants who did not reduce IFC after 2 years exhibited higher liver stiffness (from 5.05 ± 1.36 to 6.04 ± 1.90), whereas no significant changes were observed in the responder group (from 5.08 ± 1.67 to 5.76 ± 1.34).

Table 1 summarizes the anthropometric, clinical, haematological, and lifestyle characteristics of MAFLD patients, categorized into responder and non-responder groups based on the degree of improvement in IFC following a two-year intervention. Participants who responded significantly reduced their weight and BMI after the intervention period, while all participants, regardless of the extent of IFC improvement, showed a decrease in waist circumference. Although no changes were observed in systolic blood pressure in either group, diastolic blood pressure was higher after 2 years among patients who did not respond to an improvement in IFC. Only responders showed a reduction in total cholesterol, ferritin, uric acid, AST, ALT, and GGT levels, as well as an increase in HDL-c after the two-year intervention. No significant changes were observed in other analysed clinical characteristics. Regarding haematological parameters, participants who improved their IFC displayed lower concentrations of erythrocytes and eosinophils after 2 years. Additionally, both groups demonstrated a reduction in monocyte levels. Responders and non-responders to IFC improvement showed greater adherence to the MedDiet and decreased energy intake, while higher VO_2_ max was observed only in participants who achieved a reduction in their IFC after the 2-year intervention.

Table 2 shows the results of oxidative stress and inflammatory biomarkers in plasma and erythrocytes for the two groups. Non-responders, after two years of intervention, exhibited increased catalase (CAT) and superoxide dismutase (SOD) activity in erythrocytes compared to responders to the intervention. Additionally, these activities were significantly higher than the group that responded and improved their IFC. MDA levels in both erythrocytes and plasma decreased significantly in the responder group after two years of intervention. In the non-responder group, MDA tended to increase, although the differences were only significant in erythrocytes. These contrasting changes resulted in significant differences between the two groups in both erythrocytes and plasma after the intervention. TNFα levels were reduced significantly after 2 years in participants who achieved a reduction in their IFC. No differences were observed in the percentage of protein carbonyl, IL-6, and TGF-β1 levels regarding groups and timeline.

**Figure 3:**
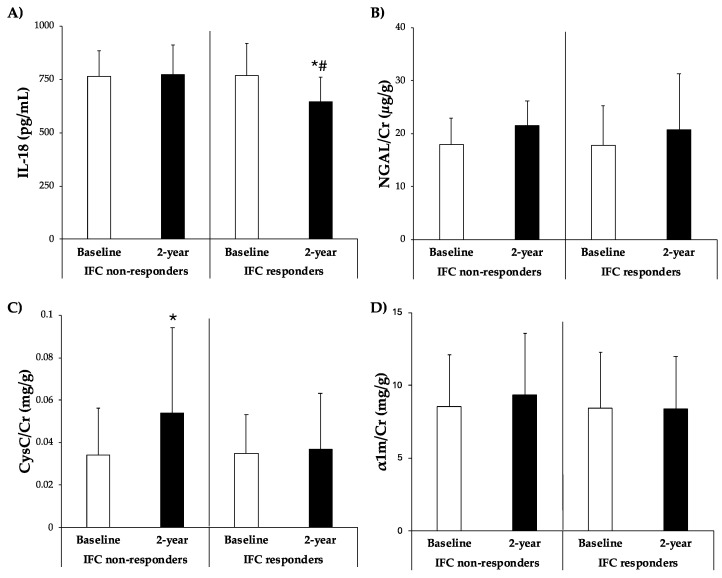
(**A**) IL-18 levels in plasma and (**B**) NGAL/Cr, (**C**) CysC/Cr, and (**D**) α1m/Cr levels in urine classified according to improvement of IFC group after 2-year intervention. Results are presented as mean ± SD. Two-way analysis of co-variance (ANCOVA) after adjustments by age and sex. * Difference in means between participants over time (baseline and 2 years). # Difference in means between groups (IFC responders and IFC non-responders). Data points are significant when *p* < 0.05.

Figure 3 illustrates the plasma levels of IL-18 and urine levels of CysC/Cr, NGAL/Cr, and α1m/Cr. The IL-18 levels were significantly lower after 2 years in participants who responded and reduced their IFC. Conversely, CysC/Cr levels were significantly higher in participants who did not respond after 2 years. There were no significant differences in the levels of NGAL/Cr and α1m/Cr between groups. 

As shown in Table 3, after 2 years of intervention, participants who did not succeed in improving their intrahepatic fat percentage had significantly increased serum creatinine levels, along with showing higher MDRD levels. It was observed, albeit not significantly, that these participants also exhibited increased CKD-EPI values. Conversely, the group of participants who did manage to decrease their IFC also succeeded in reducing serum creatinine, MDRD, and CKD-EPI levels. Moreover, they decreased UACR levels. No changes were observed in serum albumin, urine albumin, and urine creatinine levels throughout the study between groups.

## 4. Discussion

The primary outcome of the current study reveals that patients with MAFLD who decreased their IFC after a 2-year intervention with a healthy diet and lifestyle improved parameters related to cardiovascular, hepatic, renal, and inflammatory health, which represents an improvement in general health status.

Liver steatosis, characterized by the excessive and pathological accumulation of fat within liver cells, is a histologic hallmark and a potential pathogenic factor in MAFLD [40]. The reduction in the accumulation of this fat in participants who responded to the lifestyle intervention after the 2-year intervention was achieved through significantly lower IFC values compared to their baseline and compared to participants who did not respond to the intervention after the 2-year period. In fact, those who did not respond showed a small increase in IFC without it being significant. The degree of liver fibrosis can be determined using elastography techniques. These techniques measure the effect of collagen on liver stiffness, as tissue stiffness increases with higher collagen concentrations, acting as a biomarker for fibrosis deposition [41]. The current results revealed that responder participants did not show significant changes in liver stiffness, while those who did not respond experienced an increase in this parameter after 2 years. However, unlike what other studies have shown, the current participants did not reduce hepatic stiffness as other authors have reported after an intervention based on a MedDiet [42]. This disparity could be attributed to the current participants at the baseline of the study with mean values of 5.05 and 5.08 kPa, respectively, falling within the F0–F1 fibrosis stages, whereas previous studies typically involved patients with higher baseline values in the F2 fibrosis stage [43]. Moreover, over a mean of 3.6 years, greater liver stiffness was linked to a ~2.1-fold increased risk of all-cause death [44]. In fact, a changed mechanical liver phenotype—in which stiffness was exclusively connected to organ failure and served as a diagnostic marker—was linked to the start and progression of MAFLD in vivo. More accurately than blood indicators, liver stiffness was shown to be useful in distinguishing the different phases of liver fibrosis in vivo [45].

Waist circumference is a marker of abdominal fat deposition and, particularly, visceral adipose tissue [46]. Although all participants managed to significantly reduce their waist circumference, only those who reduced their IFC were those who significantly decreased their BMI after the 2-year lifestyle intervention. The change in abdominal adipose tissue, not entirely captured by BMI, may explain the weakened association among older populations. Abdominal obesity, without general obesity, showed a higher association with cardiovascular diseases than general obesity without abdominal obesity [47]. It was described that patients who lost more than 5% of body weight already showed a larger decrease in MAFLD. Moreover, a more than 10% weight loss led to further reductions in MAFLD. Additionally, 90% experienced a resolution of NASH, while 45% showed a regression of fibrosis [48]. After 2 years, individuals who did not respond to the intervention and failed to reduce their IFC showed slightly higher diastolic blood pressure, although within the normal range, suggesting a potential link between non-response and adverse cardiovascular outcomes. This result appears to be consistent with a previous study that demonstrated a relationship between increased IFC and higher levels of diastolic blood pressure [4]. However, no differences were found in current systolic blood pressure. 

Regarding general biochemistry, after the two-year intervention, only responders exhibited lower levels of total cholesterol, ferritin, AST, ALT, and GGT, along with higher levels of HDL-c. These findings align with prior research, demonstrating that improvements in IFC result in reduced ALT and GGT levels after a 6-month intervention [49]. Similarly, a meta-analysis of dietary interventions focusing on Mediterranean and hypocaloric diets also supports the improvement in transaminase levels [50]. Furthermore, lifestyle interventions involving hypocaloric diets and exercise were shown to effectively reduce IFC, triglyceride, and total cholesterol levels while increasing HDL-c levels [51]. Iron metabolism is relevant since the liver is the main reservoir associated with ferritin. If iron accumulates excessively in the liver due to some type of metabolic dysfunction, it can contribute to oxidative stress through the Fenton reaction and damage the DNA and proteins of liver cells [52]. Through the nuclear factor κB (NFκB) cascade response, ferritin can also directly activate hepatic stellate cells and contribute to hepatic fibrosis [53]. The current findings also confirmed previous results where serum ferritin levels were higher in patients with MAFLD compared to those without MAFLD, and they were associated with the risk of MAFLD in both genders [54]. Additionally, participants who reduced their IFC also exhibited a reduction in uric acid levels. This aligns with prior studies reporting higher uric acid levels in individuals with MAFLD compared to those without the condition [55].

Current participants with better IFC reduced amounts of erythrocytes and eosinophils in haematological parameters after the 2-year intervention. These outcomes are in accordance with previous studies that evidenced a link between high erythrocyte counts and increased risk of incidence and progression of MAFLD [56], while eosinophils, along with type 2 cytokines, were implicated in both tissue repair and the regeneration of hepatic tissues and in the development of fibrosis [57]. In addition, a decrease in monocyte count was observed in both groups, in which participants increased adherence to the low-calorie MedDiet. Significant associations between diet quality and monocyte count were previously reported [58], suggesting a possible beneficial effect of dietary intervention on modulating the immune system, independent of changes in IFC. However, more studies would be needed to confirm this relationship and better understand the underlying mechanisms involved.

A recent systematic review and meta-analysis highlighted the Mediterranean low-calorie diet as a favourable dietary approach for individuals with MAFLD and obesity [59]. This dietary pattern was associated with improved liver enzyme profiles in MAFLD patients. Additionally, it was suggested that it may contribute to a reduction in IFC and overall liver health [59]. However, despite both current responders and non-responders to IFC improvement exhibiting higher adherence to the MedDiet and lower calorie consumption, only the responder group demonstrated a significant increase in VO_2_ max after the 2-year intervention period. Some studies showed a relationship between physical activity and food quality, which may be more effective in reducing MAFLD than diet alone [60,61]. In fact, some of them indicated that cardiorespiratory fitness serves as a robust and independent predictor of reduced liver fat during a lifestyle intervention [62].

The current results revealed that an increased hepatic steatosis is associated with high oxidative stress-related parameters. CAT and SOD activities in erythrocytes were increased in participants who did not decrease their IFC. The results obtained seem to be in line with a previous study demonstrating a positive correlation between hepatic steatosis severity and erythrocyte SOD activities in patients with MAFLD [63]. Those authors suggested that this increase may indicate the compensatory response to increased reactive oxygen species (ROS) production derived from the situation of lipotoxicity associated with MAFLD. MDA, which represents the peroxidation of polyunsaturated fatty acids by ROS, is a well-known marker of oxidative stress. Among responders, erythrocyte MDA levels decreased, indicative of reduced oxidative stress, while non-responders exhibited elevated MDA levels, suggestive of heightened oxidative stress. Erythrocytes are a type of cell that is very susceptible to stress factors due to their absence of a nucleus, which limits their ability to renew damaged elements [64]. The observed rise in oxidative stress, evidenced by increased MDA levels, suggests a more burdensome and proinflammatory milieu. Lower levels of erythrocyte MDA were previously linked to greater adherence to the MedDiet in previous studies [65,66]. However, only responders exhibited a decline in plasma MDA levels, and these values were significantly lower than non-responder participants. A meta-analysis has proposed that the MedDiet, rich in polyphenols from olive oil, a hallmark of the diet, may contribute to the reduction in plasma MDA levels, thereby bolstering evidence for the diet’s cardioprotective effects [67].

Adipose tissue is classified as an endocrine organ since it secretes adipokines and myokines. These chemicals were seen to play a role in inflammation related to obesity and MAFLD. The over-expansion of adipose tissue leads to macrophage infiltration and increased levels of proinflammatory cytokines, such as IL-6 and TNFα [68]. In the current study, only participants who reduced IFC after the intervention had decreased TNFα levels. These changes were associated with significant weight reductions in adipose tissue and with insulin regulation related to the onset and progression of MAFLD [69]. The lack of changes in IL-6 may result from the fact that MAFLD patients showed subclinical inflammation, where IL-6 might not be significantly altered or might fluctuate within a range that did not register as a notable change in response to the intervention. High levels of TGF-β1, also classified as a proinflammatory cytokine, were observed to correlate with the severity of NAFLD, suggesting its potential as a fibrosis marker. Moreover, it was implicated as playing a crucial role in myofibroblast activation, contributing to liver cirrhosis [70]. No differences were observed among the current participants between groups and timepoints. This could be because the current participants at baseline did not have fibrosis, and even though the group that did not show improvement in IFC after a 2-year period did increase liver fibrosis, the values remained within the non-pathological stage, potentially explaining the lack of significant changes in TGF-β1 plasma levels.

In addition to addressing improvements in fatty liver, it is crucial to consider the interrelation with other conditions such as CKD. In this regard, it was observed that individuals who experienced IFC improvements also showed enhancements in renal function and some general renal filtration markers. The current findings are consistent with a previous study, which demonstrated a significant association between high intrahepatic triglyceride content and increased risk of CKD [71]. The MDRD and CKD-EPI equations are commonly used to estimate GFR. The current study found that participants with MAFLD who did not show improvement in IFC after the 2-year intervention had higher MDRD levels, whereas those who achieved improvement had lower MDRD and CKD-EPI levels. These findings are consistent with previous studies that have linked the severity of MAFLD with the prevalence of CKD [16,23]. UACR levels were also observed to significantly decrease in the current MAFLD patients who improved their IFC. An investigation reported that MAFLD subjects showed a significantly higher prevalence of UACR compared to those without MAFLD, thus associating intrahepatic triglyceride contents with UACR [71]. Participants who successfully reduced their IFC over the two-year intervention period demonstrated a decrease in serum creatinine values, whereas those who did not achieve this reduction showed increased serum creatinine levels. These findings corroborate previous research indicating high serum creatinine levels in individuals with MAFLD compared to those without MAFLD [72]. Finally, the current findings from correlations demonstrate that as IFC reduces, there is a corresponding rise in MDRD and serum creatinine values.

IL-18, besides its inflammatory role, emerges as a biomarker for kidney injury, with high serum levels associated with conditions like tubular necrosis and delayed graft function, indicating its involvement in kidney pathology induced by diverse disease processes, including urinary obstruction [73]. The current study reveals a notable trend since participants who failed to improve their IFC over the 2-year intervention exhibited increased IL-18 plasma levels, while those who succeeded experienced significant reductions compared to the other group. This finding aligns with previous research demonstrating high serum IL-18 levels correlating with the progression of MAFLD and advancing fibrosis [74]. The relationship between plasma IL-18 levels and MAFLD progression, coupled with its association with renal injury, suggests the potential of IL-18 as a valuable biomarker in managing these metabolic conditions.

Similarly, CysC, a cysteine proteinase inhibitor protein, is consistently produced by all nucleated cells and undergoes efficient filtration by the kidneys. Predominantly reabsorbed and metabolized in the proximal tubule, it exhibits minimal excretion in urine [75]. Urinary CysC levels closely correlate with both the early stages and progression of CKD, making it an optimal endogenous marker for assessing renal function impairment [76]. The current results revelated that patients who did not improve their IFC after the 2-year intervention had significantly increased CysC/Cr levels. Some researchers suggested that the urinary CysC/Cr ratio could serve as a risk factor for cardiovascular disease (CVD) and CKD in patients with obesity and MetS [77].

Urinary alpha-1-microglobulin (α1m) is a tubular protein commonly utilized as a biomarker for acute lesions in the proximal tubules [78]. A cross-sectional study demonstrated a significant correlation between urinary α1m levels and MAFLD, suggesting its potential as a non-invasive diagnostic biomarker and a screening tool for identifying advanced fibrosis. Furthermore, these findings indicated that urinary α1m could also contribute to the early detection of kidney injury beyond eGFR [79]. However, the current study did not find significant changes in α1m/Cr levels in MAFLD patients following an intervention aimed at reducing IFC. This outcome may be expected, as these patients typically exhibit normal or only slightly reduced filtration rates, which could explain the absence of substantial changes. 

High levels of Neutrophil gelatinase-associated lipocalin (NGAL), a secretory protein released by activated neutrophils, have been identified as an independent predictor not only of kidney complications but also of cardiovascular and liver-related diseases [80]. The current study reveals a small increase in NGAL urine levels over the course of 2 years; however, this increase did not reach statistical significance. Moreover, the renal impairment manifested by the current participants remained notably subtle when contrasted with the higher cases of CKD documented in the existing literature [81]. These observations hint at a more intricate interplay between NGAL levels and renal dysfunction, diverging from the straightforward relationship observed in severe CKD cases. 

## 5. Strengths and Limitations of the Study

The main strength of the current study is its comprehensive assessment of both IFC and biomarker levels related to MAFLD and CKD. By including a diverse set of biomarkers and employing rigorous methodology, the current study provides a robust foundation for understanding the potential association between MAFLD and CKD. The longitudinal nature of the two-year intervention allows for insights into the effectiveness of dietary and lifestyle modifications in mitigating both hepatic and renal impairments. 

The primary constraint of this research lies in the relatively modest sample size. However, it is noteworthy that despite this limitation, the sample size was enough to reveal differences in biomarker levels between the group with higher IFC and the group with lower IFC, showing a potential association between MAFLD and CKD. It is worth noting that both groups had an equal distribution of sexes. That no liver biopsies were performed could be a limitation of this study. Still, MRI has been used for IFC evaluations, which is a well-recognized, trustworthy, and non-invasive method that greatly lowers danger for patients.

## 6. Conclusions

The current study demonstrates that patients with MAFLD undergoing a two-year intervention with a low-calorie Mediterranean diet experienced an IFC reduction and, if successful, exhibited improvements in parameters associated with renal dysfunction. These findings suggest that dietary interventions targeting MAFLD could potentially have a beneficial impact on kidney health. The current results support the notion that MAFLD contributes to the pathophysiology of CKD, emphasizing the importance of closely monitoring MAFLD patients for the development of CKD.

## Figures and Tables

**Table 1:** Characteristics of participants with MAFLD according to IFC improvement at baseline and after 2-year intervention.

	IFC Non-Responders		IFC Responders		*p*-Value
	Baseline (n = 20)	2-Year Change (n = 20)	Baseline (n = 20)	2-Year Change (n = 20)	
Anthropometry					
Weight (kg)	91.5 ± 11.3	90.0 ± 11.6	94.2 ± 17.2	90.6 ± 15.2 *	0.182
BMI (kg/m^2^)	33.6 ± 3.97	32.9 ± 3.79	34.2 ± 4.62	32.7 ± 5.01 *	0.637
Waist circumference (cm)	113.3 ± 8.59	110.3 ± 9.13 *	110.8 ± 9.38	106.9 ± 9.57 *	0.628
Systolic BP (mmHg)	134.2 ± 11.8	133.3 ± 11.8	137.2 ± 21.7	137.5 ± 22.8	0.858
Diastolic BP (mmHg)	78.5 ± 7.28	82.4 ± 7.15 *	82.2 ± 9.18	84.6 ± 11.2	0.358
Clinical parameters					
Glucose (mg/dL)	122.3 ± 41.0	120.9 ± 37.8	107.1 ± 17.5	103.4 ± 22.2	0.603
HbA1c (%)	6.21 ± 1.01	6.14 ± 0.87	5.90 ± 0.68	5.82 ± 0.64	0.866
Cholesterol total (mg/dL)	204.1 ± 44.1	213.6 ± 53.7	212.6 ± 36.2	196.8 ± 43.4 *#	0.002
HDL-c (mg/dL)	42.1 ± 4.82	43.3 ± 8.04	42.6 ± 7.54	44.7 ± 8.78 *	0.442
LDL-c (mg/dL)	121.1 ± 35.5	123.5 ± 39.7	135.9 ± 36.5	125.8 ± 34.3	0.114
Triglycerides (mg/dL)	186.7 ± 78.3	187.5 ± 86.3	169.4 ± 57.4	154.6 ± 55.9 #	0.349
CRP (mg/dL)	0.587 ± 0.754	0.511 ± 0.485	0.566 ± 0.504	0.491 ± 0.509	0.968
AST (U/L)	24.9 ± 16.5	26.9 ± 13.0	32.7 ± 23.5	23.8 ± 7.54 *#	0.045
ALT (U/L)	35.1 ± 27.9	38.0 ± 24.8	41.7 ± 19.1	32.2 ± 11.6 *#	0.026
GGT (U/L)	59.4 ± 59.6	59.1 ± 42.7	56.1 ± 85.6	32.6 ± 16.4 *	0.346
Serum ferritin (ng/mL)	118.8 ± 106.4	118.3 ± 115.3	127.1 ± 131.2	70.1 ± 36.5 *	0.095
Uric acid (mg/dL)	6.25 ± 1.73	6.33 ± 1.50	6.29 ± 0.96	5.82 ± 1.11 *	0.082
Haematological parameters					
Haematocrit (%)	42.9 ± 3.4	42.7 ± 3.4	44.3 ± 4.2	43.6 ± 3.4	0.293
Erythrocytes (10^6^/μL)	4.79 ± 0.25	4.73 ± 0.25	4.93 ± 0.40	4.81 ± 0.30 *	0.163
Leukocytes (10^3^/μL)	8.29 ± 1.56	7.96 ± 2.01	6.69 ± 1.66 #	6.46 ± 1.26 #	0.717
Platelets (10^3^/μL)	248.8 ± 43.0	236.1 ± 43.2	236.1 ± 43.2	223.6 ± 46.4	0.729
Neutrophils (10^3^/μL)	4.55 ± 1.04	4.27 ± 1.47	3.70 ± 1.17 #	3.51 ± 0.96	0.714
Lymphocytes (10^3^/μL)	2.73 ± 0.61	2.70 ± 0.62	2.19 ± 0.56 #	2.24 ± 0.42 #	0.299
Monocytes (10^3^/μL)	0.755 ± 0.356	0.650 ± 0.257 *	0.522 ± 0.143	0.485 ± 0.121 *	0.418
Eosinophils (10^3^/μL)	0.262 ± 0.135	0.274 ± 0.162	0.221 ± 0.176	0.166 ± 0.081 *#	0.066
Basophils (10^3^/μL)	0.063 ± 0.024	0.070 ± 0.026	0.055 ± 0.023	0.062 ± 0.027	0.617
Lifestyle parameters					
Energy intake (kcal/day)	2249 ± 759	1694 ± 535 *	2336 ± 696	1767 ± 448 *	0.775
MedDiet (17-score)	7.80 ± 3.04	11.8 ± 1.88 *	8.70 ± 2.66	12.0 ± 3.07 *	0.747
Chester step test (VO_2_ max)	30.2 ± 7.4	29.1 ± 9.2	33.8 ± 9.9	39.2 ± 13.5 *#	0.073

Abbreviations: BMI: body mass index; systolic BP: systolic blood pressure; diastolic BP: diastolic blood pressure; HbA1c: glycated haemoglobin A1c; HDL-c: high-density lipoprotein; LDL-c: low-density lipoprotein; AST: aspartate aminotransferase; ALT: alanine aminotransferase; GGT: gamma glutamyl transferase; CRP: c-reactive protein; MedDiet: Mediterranean diet; SD: standard deviation. Results are expressed as mean ± SD. Two-way analysis of co-variance (ANCOVA) after adjustments by age and sex. * Difference in means between participants over time (baseline and 2 years). # Difference in means between groups (IFC responders and IFC non-responders). Data points are significant when *p* < 0.05.

**Table 2:** Oxidative stress and inflammatory biomarkers in plasma and erythrocytes of participants with MAFLD according to IFC improvement at baseline and after 2-year intervention.

	IFC Non-Responders		IFC Responders		*p*-Value
	Baseline (n = 20)	2-Year Change (n = 20)	Baseline (n = 20)	2-Year Change (n = 20)	
Antioxidant biomarkers					
CAT activity (k/10^9^ erythrocytes)	12.5 ± 2.58	16.0 ± 1.61 *	12.5 ± 1.76	12.9 ± 1.47 #	<0.001
SOD activity (pkat/10^9^ erythrocytes)	7.72 ± 0.49	8.96 ± 0.37 *	7.80 ± 0.48	7.88 ± 0.45 #	<0.001
Oxidative damage					
MDA (ng/10^9^ erythrocytes)	10.3 ± 1.16	11.2 ± 0.81 *	10.1 ± 0.82	9.55 ± 0.70 *#	<0.001
MDA (ng/L plasma)	1.61 ± 0.64	2.01 ± 0.96	1.85 ± 0.79	1.25 ± 0.69 *#	0.005
Protein carbonyl (%)	100 ± 58.7	110.7 ± 52.1	98.0 ± 75.0	99.9 ± 36.6	0.901
Protein levels					
IL-6 (pg/mL)	4.23 ± 0.25	4.28 ± 0.48	4.16 ± 0.28	4.08 ± 0.28	0.282
TNFα (pg/mL)	3.87 ± 0.40	3.86 ± 0.40	3.91 ± 0.76	3.65 ± 0.46 *	0.145
TGF-β1 (ng/mL)	2.23 ± 1.01	2.16 ± 1.46	2.74 ± 1.40	2.59 ± 1.60	0.781

Abbreviations: CAT: catalase, SOD: superoxide dismutase, MDA: malondialdehyde, IL-6: interleukin-6, TNFα: tumour necrosis factor alpha, TGF-β1: transforming growth factor-β1, SD: standard deviation. Results are expressed as mean ± SD. Two-way analysis of co-variance (ANCOVA) after adjustments by age and sex. * Difference in means between participants over time (baseline and 2 years). # Difference in means between groups (IFC responders and IFC non-responders). Data points are significant when *p* < 0.05.

**Table 3:** Kidney function parameters of participants with MAFLD according to IFC improvement at baseline and after 2-year intervention.

	IFC Non-Responders		IFC Responders		*p*-Value
	Baseline (n = 20)	2-Year Change (n = 20)	Baseline (n = 20)	2-Year Change (n = 20)	
MDRD (mL/min/1.73 m^2^)	90.4 ± 10.3	94.1 ± 8.9 *	93.6 ± 8.4	83.7 ± 9.5 *#	<0.001
CKD-EPI (mL/min/1.73 m^2^)	91.8 ± 15.0	94.7 ± 12.3	95.8 ± 11.8	85.0 ± 16.7 *#	<0.001
Serum albumin (g/L)	4.36 ± 0.24	4.36 ± 0.27	4.35 ± 0.22	4.30 ± 0.20	0.291
Serum creatinine (mg/dL)	0.759 ± 0.111	0.837 ± 0.154 *	0.790 ± 0.121	0.756 ± 0.114 *#	<0.001
Urine albumin (mg/L)	11.1 ± 8.9	6.45 ± 8.14	9.0 ± 10.9	6.13 ± 4.31	0.723
Urine creatinine (mg/L)	121.8 ± 51.8	116.8 ± 44.9	115.6 ± 56.6	123.4 ± 48.7	0.558
UACR (mg/g)	8.87 ± 5.94	5.51 ± 5.03	8.15 ± 9.31	5.30 ± 3.73 *	0.980

Abbreviations: MDRD: modification of diet in renal disease, CKD-EPI: chronic kidney disease epidemiology collaboration, UACR: urine albumin-to-creatinine ratio, SD: standard deviation. Results are expressed as mean ± SD. Two-way analysis of co-variance (ANCOVA) after adjustments by age and sex. * Difference in means between participants over time (baseline and 2 years). # Difference in means between groups (IFC responders and IFC non-responders). Data points are significant when *p* < 0.05.

## Data Availability

There are restrictions on the availability of data for this trial due to the signed consent agreements around data sharing, which only allow access to external researchers for studies following the project purposes. Requestors wishing to access the trial data used in this study can make a request to pep.tur@uib.es.
